# Genomic Differences Between Nervous Necrosis Virus (NNV) Reassortants Isolated From Wild and Farmed Fish: Implications for Viral Fitness, Temperature Adaptation and Virulence

**DOI:** 10.1155/tbed/8896753

**Published:** 2025-06-18

**Authors:** Lucía Vázquez-Salgado, José G. Olveira, María Jurado-Rodeyro, Carlos P. Dopazo, Isabel Bandín

**Affiliations:** Aquatic One Health Research Center (ARCUS), Universidade de Santiago de Compostela (USC), Santiago de Compostela 15782, Spain

**Keywords:** betanodavirus, host specificity, mutations, NCR, reassortant, replication, temperature, virulence

## Abstract

Nervous necrosis virus (NNV), one of the most widespread fish pathogens, is classified into four genotypes: Barfin flounder-, Redspotted grouper-, Striped Jack- and Tiger puffer NNV (BFNNV, RGNNV, SJNNV and TPNNV, respectively), which show different thermotolerance and geographical distribution. Reassortant RGNNV/SJNNV strains are detected in Southern Europe, associated to disease outbreaks in Senegalese sole and gilthead seabream larvae or early juveniles, with water temperatures around 22–23°C. These strains contain amino acid changes in the capsid and polymerase protein when compared with the reference strains of each genotype. We have assessed the effect of temperature on the replicative fitness of four reassortants obtained from wild pilchard and mackerel and their pathogenic potential against sole and turbot. In vitro replication assays showed an improved replication of the mackerel isolate at 15°C while it was delayed at 20 and 25°C. Substitutions in the viral polymerase, particularly Arg237, and differences in the non-coding regions (NCR) might account for this adaptation to replicate at a suboptimal temperature for reassortants. In addition, in the in vivo assays at different temperatures, the mackerel isolate caused the lowest mortality and showed limited replication in sole brain tissue. However, in the experimental infection in turbot at 15°C, it displayed an exponential replication, although it did not cause mortality. The analysis of the capsid protein (Cp) of this isolate points to position 237 as a putative host specificity determinant that might favour the interaction with turbot cell receptors. In conclusion, substitutions observed in the mackerel strain suggest an adaptation to replicate at low temperature, which would enable it to spread to the cold waters of the North Atlantic Ocean. In addition, it also highlights the potential risks associated with the introduction of NNV strains from the wild into fish farms or new areas.

## 1. Introduction

Worldwide production of marine fish species such as sea bass (*Dicentrarchus labrax*), grouper (*Epinephelus* sp.), or sole (*Solea* spp.) is severely threatened by the nervous necrosis virus (NNV; Genus *Betanodavirus*), the etiological agent of the viral encephalopathy and retinopathy (VER) [1]. NNV is a small and naked virus whose genome is organised into two molecules of positive sense and single-stranded RNA, namely RNA1 and RNA2. RNA1 codes for the RNA-dependent RNA-polymerase (RdRp), whereas RNA2 encodes the capsid protein (Cp) [2]. During the infectious process, a third RNA molecule (RNA3) is synthetised from the 3′end of RNA1 [3]. According to Nishizawa et al. [4], the current NNV classification is based on a partial sequence of the RNA2 that differentiates 4 genotypes: Barfin flounder-, Redspotted grouper-, Striped Jack- and Tiger puffer NNV (BFNNV, RGNNV, SJNNV and TPNNV, respectively). BFNNV strains have been detected in cold water fish species in Northern Europe, America and Japan (4–15°C), while RGNNV are associated with mortality episodes in tropical and temperate species along the Mediterranean basin, Asia, and Oceania (23–30°C). SJNNV strains have been isolated in Japan and the Iberian Peninsula at mild temperatures (20–25°C), and TPNNV has been described once in Japan [1]. In 2007, Toffolo et al. [5] reported natural reassortment between both genomic segments of RGNNV and SJNNV genotypes. The most frequently detected combination is RNA1-RGNNV type/RNA2-SJNNV type and is associated with severe disease outbreaks in larvae and early juveniles of Senegalese sole (*Solea senegalensis*) and gilthead seabream (*Sparus aurata*) in Southern Europe [6, 7]. These reassortant strains harbour mutations in both RNA molecules compared to the original genotypes [8–11] and could have emerged as part of an evolutionary mechanism commonly displayed by segmented RNA viruses that introduce mutations into the genetic material to broaden the host range [12].

Recently, we have reported the isolation of four RGNNV/SJNNV strains from wild fish caught in natural shoals off the northwestern Atlantic coast of Spain at an average water temperature of 16°C [13], which is below the temperature considered suitable for the replication of the reassortants [14]. In addition, these isolates showed several nucleotide (nt) and amino acid (aa) differences regarding other reassortants isolated from farmed fish.

Climate change is affecting wild and farmed fish population dynamics [15–17]. RNA viruses, like NNV, seem to adapt easily to environmental changes as shown by the increase in viral zoonotic diseases [18, 19] but their ability to adapt to changing temperatures in aquatic environments has been poorly analysed. Therefore, the objective of the present study was to assess the effect of temperature on the replicative fitness of these 4 wild RGNNV/SJNNV strains, both in vitro and in vivo, as well as on the pathogenic potential against the main marine fish species farmed in the area (sole and turbot).

## 2. Materials and Methods

### 2.1. Viral Strains and Propagation in E-11 Cell Line

Five RGNNV/SJNNV reassortants were used: four wild isolates obtained from pilchard (SpSpIAUSC756.19, SpSpIAUSC939.20 and SpSpIAUSC997.21, hereafter Sp756.19, Sp939.20 and Sp997.21, respectively) and mackerel (SpSscIAUSC1002.21, hereafter Ssc1002.21 [13]), and one obtained from farmed sole (SpSsIAUSC160.03, hereafter Ss160.03 [6]) used as a reference strain.

Viruses were propagated at a multiplicity of infection (MOI) of 0.1 in E-11 cell monolayers grown in 75 cm^2^ flasks containing Leivobitz's 15 medium (L-15; Gibco) supplemented with 5% foetal bovine serum (FBS; Hyclone Laboratories, Inc.). After a 1-h adsorption, viral inoculum was discarded and monolayers were incubated with L-15 supplemented with 2% FBS (maintenance medium) at 25°C, until the cytopathic effect (CPE) was complete. Crude viruses were clarified by centrifugation at 4000 × *g* for 20 min at 4°C, and supernatants were titrated in E-11 monolayers seeded in 96-well plates using six replicate wells per dilution and the end-point dilution method. Titres were expressed as Tissue Culture Infectious Dose 50 per ml (TCID_50_/ml) according to Reed and Muench [20].

### 2.2. In Vitro Replication Assay

In vitro replication assays were carried out at 15, 20 and 25°C in E-11 monolayers seeded in 48-well plates up to 240 h. The four wild reassortants and the sole strain were inoculated in triplicate at a MOI of 0.1 for 1 h. After adsorption, the inoculum was discarded, and monolayers were washed three times with L-15 medium and covered with maintenance medium. Samples consisting of 100 µl of cell culture supernatant were collected from each biological replicate every 24 h and stored at −20°C for virological analyses (titration at 25°C and qPCR).

### 2.3. Experimental Bath Infections

Juveniles of Senegalese sole and turbot (*Scophthalmus maximus*) weighing 1 ± 0.2 g were provided by a local fish farm (Stolt Sea Farm, S.L.). Immediately upon arrival at the aquarium facilities of the University of Santiago de Compostela, the presence of potential pathogenic bacteria and viruses was ruled out following the procedure described by Olveira et al. [21]. Fish handling was conducted in strict accordance with the European Union Guidelines (Directive 2010/63/UE) for the protection of animals used for scientific purposes, and the infection protocol was approved by the Galician Committee for experimental animal welfare and by the Xunta de Galicia (Permit Id. 15.010/2020/004).

Fish were acclimated at 15°C ± 1°C (temperature of the facility) in 300 l tanks for a week, and then each fish species was randomly distributed in 50 l opaque tanks (30 fish per tank). For some sole infections the water temperature was progressively increased at a rate of 1°C/day, up to 18 ± 1°C, or 22 ± 1°C.

**Figure 1:**
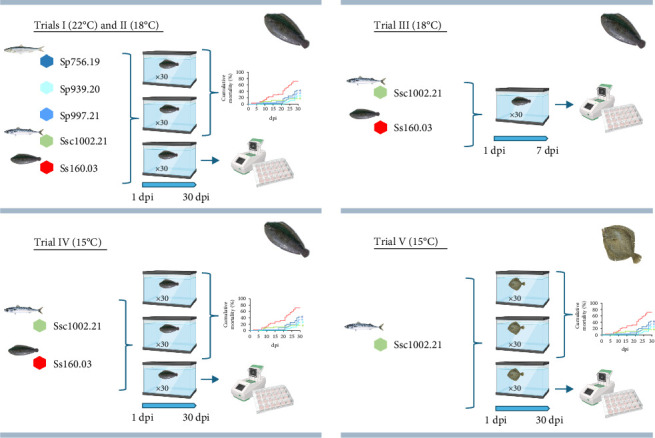
Schematic representation of the five fish infection trials performed at 22, 18 and 15°C in sole and at 15°C in turbot, with the four wild reassortant isolates and the sole strain.

Five infection trials were performed by immersion during 3 h using a viral load of 10^5^ TCID_50_/ml (Figure 1) to assess the pathogenic potential of the different NNV strains at different temperatures:

• Trials I and II: sole juveniles were infected with each wild isolate and the sole Ss160.03 strain for 30 days at 22°C and at 18°C, respectively.

The subsequent two experimental infections were performed with the mackerel isolate and the sole strain.

• Trial III: sole juveniles were infected at 18°C for 7 days.
• Trial IV: sole juveniles were infected at 15°C for 30 days.

The last experimental infection (trial V) was performed only with the mackerel isolate. Turbot juveniles were infected at 15°C for 30 days.

Three replicate tanks (*n* = 30) were established per condition except for Trial III, in which only one tank was set up per viral strain to reduce the number of fish used and because according to Trial II, no mortalities were recorded at 18°C during the first 7 days post infection (dpi). A negative control tank bathed with L-15 culture medium was established for each temperature and handled as fish from the experimental infections. Fish were supervised daily to remove dead fish and to monitor the appearance and severity of clinical signs.

Mortality curves were estimated in Trials I, II, IV and V by pooling data from the two replicate tanks. Three fish from one tank per condition were randomly sampled (weekly in Trials I, II, IV and V, and daily in Trial III) and euthanised using an MS-222 overdose. The head region was aseptically collected, mechanically homogenised in Earle's balanced salt solution (Hyclone Laboratories, Inc.), supplemented with antibiotics (amphotericin B 200 µg/ml, gentamycin 500 µg/ml, penicillin 1000 units/ml, and streptomycin 1000 units/ml), and then centrifuged at 4000×*g* for 20 min at 4°C. Supernatants were collected and stored at −20°C for virological analysis (virus isolation in E-11 at 25°C and qPCR).

### 2.4. Virological Analysis

Supernatants of homogenised fish brains were inoculated in duplicate in E-11 cell monolayers seeded in 48-well plates at 25°C for 7–10 days for virus isolation. Then, cells were scrapped and inoculated onto new E-11 monolayers for an additional 10 days. Non-infected cells were used as controls. Samples showing CPE were subjected to titration as previously described.

Total RNA was extracted from fish brain homogenates and from samples of the in vitro replication assay using the NucleoSpin Kit (Macherey-Nagel) following the instructions provided by the manufacturer. cDNA was synthesised through RNA (9 µl) incubation for 5 min at 95°C with random primers (200 nM) and then for 1 h at 42°C with the RT mixture from the RevertAid First Strand cDNA Synthesis Kit (Thermo Scientific, Inc.), in a T100 thermal cycler (Bio-Rad). The reaction was finished with polymerase inactivation at 70°C for 10 min and then cooled to 4°C.

The obtained cDNA (2 µl) was amplified by qPCR in a CFX-96 Real Time PCR Detection System (Bio-Rad) in the presence of 10 µl of BlasTaq 2X qPCR Master Mix (abm) and 200 nM of SnodR1 F/R or NodR2 F/R primers [22] when applicable, following the protocol provided by the kit, except for the annealing-extension step, conducted at 59°C (SnodR1) or 57°C (NodR2) for 20 s. Total RNA1 and RNA2 copies were estimated from two standard curves generated from 20-fold dilutions (10^7^–10^2^ RNA copies/µl) of plasmids containing RNA1 and RNA2 complete segments of Ss160.03 strain.

### 2.5. Determination of 5′ and 3′ Non-Coding Regions (NCR) of RNA1 and RNA2 Segments of Strain Ssc1002.21

The sequence of 5′ and 3′ NCR of RNA1 and RNA2 molecules of strain Ssc1002.21 was determined by rapid amplification of cDNA ends (RACE). 5′ NCR was obtained using a FirstChoice RLM-RACE kit (Ambion) according to manufacturer's guide and specific primers (RNA1 : 5′-GGTGTTATGTGCTCGCGGCTCTTT-3′; RNA2: 5′-GGCGACGACTGCACCACGAG-3′ [10],). The sequence of 3′ NCR was obtained using a SMARTer RACE kit (Clontech) with specific primers (RNA1: 5′-AGCTCTTCCAGGTGATGATGGACACG-3′; RNA2: 5′-GGATTTCGTTCCATTCTCTTGGG-3′; [23]). PCR products were purified from a 2% agarose gel, inserted in a pJET1.2/blunt cloning vector (Thermo Scientific) following the kit instructions, and transformed into DH5*α* competent cells (NZYTech). Three individual clones were sequenced using pJET fw/rv primers (provided by the kit) to determine the sequence of 5′ and 3′ ends.

Nucleotide sequences were aligned with the strains of the reference genotypes (SGWak97 for RNA1 and SJNag93 for RNA2) and the sole strain Ss160.03 (Table 1) using Geneious 7.1.3. Minimum free energy secondary structures of the NCR regions of RNA1 and RNA2 were predicted using the RNAfold Web Server with default parameters (http://rna.tbi.univie.ac.at//cgi-bin/RNAWebSuite/RNAfold.cgi).

### 2.6. Statistical Analysis

Statistical analyses were performed with GraphPad Prism 8.1. Viral load was expressed as the mean ± SD of three biological replicates at each point and analysed by two-way ANOVA with Dunnett's multiple comparisons test. The normality of the data was evaluated before performing ANOVA tests using Kolmogorov–Smirnov correction. Mortality curves were analysed using the Kaplan–Meier test, and differences were assessed with the Log-Rank (Mantel–Cox) test. Statistically significant differences were defined as *p* value < 0.05.

## 3. Results

### 3.1. Replication Assays In Vitro

**Figure 2:**
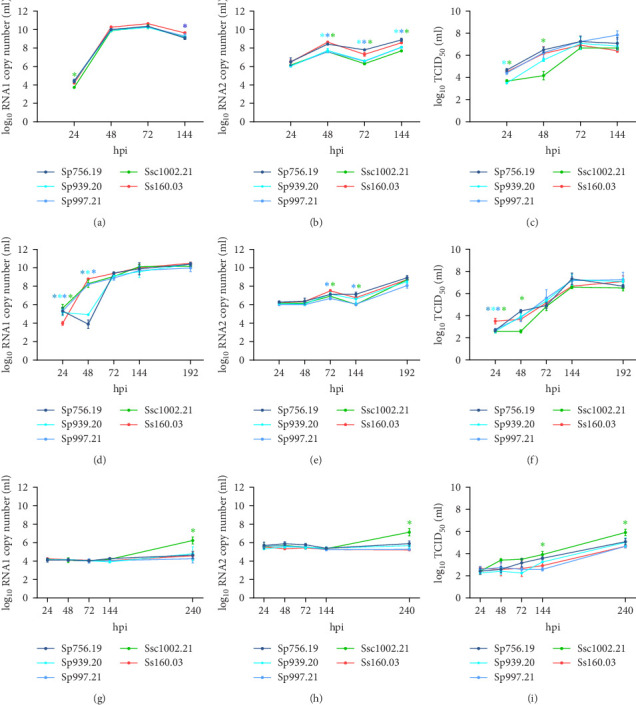
In vitro replication kinetics in E-11 of the NNV reassortants. Results of the assays performed at 25°C (A–C), 20°C (D–F), and 15°C (G–I), were expressed as log_10_ RNA1 and RNA2 copies/ml, and TCID_50_/ml (titration was performed at 25°C). Data are expressed as the mean ± SD of the three replicates per condition. Asterisks (*⁣*^*∗*^) represent statistically significant differences regarding the reference Ss160.03 strain at each time point (*p* value < 0.05). NNV, nervous necrosis virus.

At 25°C, an initial delay in the RNA 1 synthesis was recorded in the mackerel isolate with respect to the sole strain but also to the other wild strains. Thus, at 24 h post infection (hpi), this strain showed 5.37 × 10^3^ copies vs. 2.31 × 10^4^, the average copy number of the other strains (*p* value 0.001; Figure 2A). However, at the end of the experiment, this strain displayed a similar RNA1 load to that of the sole strain, whereas one pilchard isolate (Sp756.19) showed significantly lower values (*p* value 0.02). Regarding RNA2 (Figure 2B), although at 24 hpi the value was similar in all reassortants, thereafter, the mackerel strain and two of the pilchard strains (Sp939.20 and Sp997.21) showed a significantly lower load (*p* value < 0.05). An initial delay was also observed in progeny production in both the mackerel and the pilchard isolate Sp939.20 when compared with the sole strain (Figure 2C). The production of infective particles rapidly increased in the pilchard isolate, whereas it took 72 h for the mackerel isolate to reach the production level of the other strains. Complete CPE was detected at 144 hpi for all strains.

At 20°C, the initial RNA1 load of the wild reassortants was significantly higher than that of the reference sole strain Ss160.03 (*p* value < 0.05; Figure 2D). At 48 hpi, whereas the sole strain and two of the wild isolates showed an increase in the copy number, a delayed replication was observed in the pilchard strains Sp756.19 and Sp939.20. However, at the end of the experiment, similar values were recorded for all strains. Likewise, no significant differences were detected in the final RNA2 production (Figure 2E), although at 72 and 144 hpi the pilchard Sp997.21 and the mackerel isolates showed a lower replication (around 1 log). Surprisingly, viable virus production in the wild strains at 24 hpi was on average 1 log lower (4.48 × 10^2^ TCID_50_/ml) than in the reference strain Ss160.03 (3.16 × 10^3^ TCID_50_/ml; *p* value < 0.05; Figure 2F). However, at 48 hpi a delayed replication was observed only in the mackerel strain, showing values significantly lower than the Ss160.03 strain (*p* value < 0.05). By the end of the experiment (192 hpi), similar viral titres were recorded for all strains.

At 15°C, the genomic load of the wild isolates remained stable and at similar values as the sole strain (on average, 10^4^ and 5.6 × 10^5^ RNA1 and RNA2 copies/ml; respectively) until 144 hpi (Figure 2G,H). However, at 240 hpi, a significant increase was observed in the mackerel strain (1.72 × 10^6^ and 1.36 × 10^7^ copies/ml for RNA1 and RNA2, respectively, *p* value 0.02). Despite no CPE being detected in any of the infected wells at this temperature, the titration at 25°C revealed that the production of viable particles in the mackerel isolate was also significantly higher than that of the sole strain, not only at the end of the experiment (240 hpi) but also at 144 hpi (Figure 2I).

### 3.2. In Vivo Trials: Mortality Curves and Replication Kinetics

**Figure 3:**
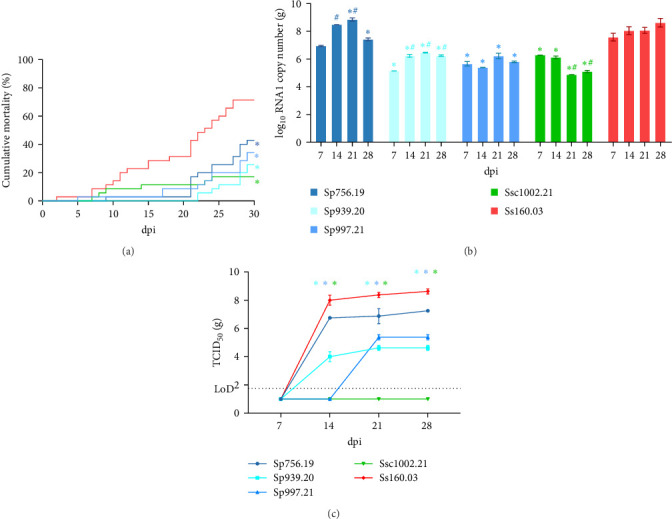
In vivo trials conducted at 22°C in Senegalese sole (Trial I). Cumulative mortality (A) and viral load expressed as log_10_RNA1 copies/ml (B) and TCID_50_/ml (C), recorded for each reassortant. Results are indicated as the mean ± SD of the three replicates per condition. Asterisks (*⁣*^*∗*^) represent statistically significant differences regarding the reference Ss160.03 strain at each time point (*p* value < 0.05). Hashtags (#) represent statistically significant differences for each strain regarding 7 dpi (*p* value < 0.05).

At 22°C (Trial I), cumulative mortality in sole provoked by the wild isolates was moderate. In the groups infected with the pilchard isolates, mortality ranged from 21 to 43%, and the lowest value (19%) was recorded in the fish challenged with the mackerel strain (Figure 3A). However, in all cases it was significantly lower than that recorded in fish infected with the sole strain (72%). Regarding viral load in brain tissue, RNA1 replication of strain Sp756.19 was similar to that of the reference strain Ss160.03 (Figure 3B), increasing from 8.97 × 10^6^ copies on day 7 pi to 7.67 × 10^8^ on day 21 post infection (pi), although on day 28 pi, RNA1 copy number dropped around 1 log. Infective particles of both strains were recovered in E-11 cells from all sampling points, but only samples from day 14 pi onwards were above the limit of detection (LoD; Figure 3C) of the endpoint dilution method. The titre of Sp756.19 was on average 1 log lower than the reference Ss160.03 (10^7^ vs. 10^8^ TCID_50_/g; *p* value > 0.05). The remaining pilchard reassortants showed moderate replication, and the average copy number throughout the experiment was around 2 logs lower than the Ss160.03 strain (10^5^ – 10^6^ vs. 10^8^ copies; *p* value < 0.05). The titre of strain Sp997.21 remained stable between days 21 and 28 pi (10^5^ TCID_50_/g), while strain Sp939.20 showed a 0.5 log increase between days 14 and 21 pi (from 10^4^ to 4.22 × 10^4^ TCID_50_/g; *p* value < 0.05). Finally, the RNA1 load of the mackerel strain Ssc1002.21 decreased during the course of the experiment, and the difference regarding the Ss160.03 strain was around 3.5 logs on days 21 and 28 pi. Although viable viral particles were recovered at all sampling points, titration values were below the LoD.

**Figure 4:**
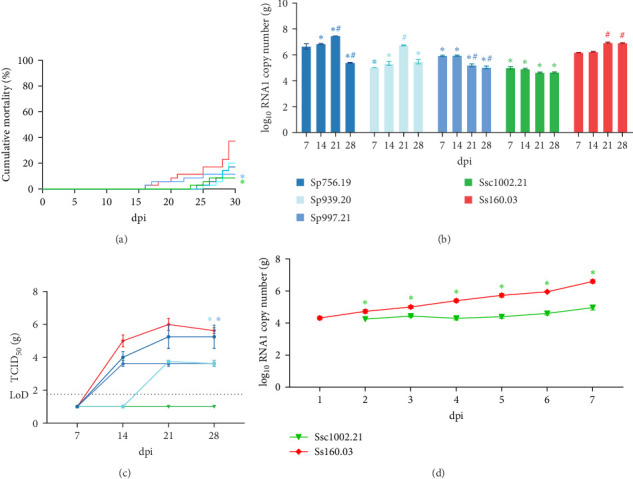
In vivo trials conducted at 18°C in Senegalese sole (Trial I and II). Cumulative mortality (A) and viral load expressed as log_10_RNA1 copies/ml (B) and TCID_50_/ml (C), recorded for each reassortant in Trial I, and for the mackerel and sole strains in Trial II (D). Results are expressed as the mean ± SD of the three replicates per condition. Asterisks (*⁣*^*∗*^) represent statistically significant differences regarding the reference Ss160.03 strain at each time point (*p* value < 0.05). Hashtags (#) represent statistically significant differences for each strain regarding 7 dpi (*p* value < 0.05).

At 18°C (Trial II), the onset of mortalities was delayed compared to what was observed at 22°C and cumulative mortality values were lower in all infected groups. The highest value (37%) was obtained in the group challenged with the sole strain (Figure 4A) and significant differences were recorded only with groups infected with the pilchard Sp997.21 and the mackerel Ssc1002.21 isolates. RNA1 of two of the pilchard strains (Sp756.19 and Sp939.21) showed an increasing replication trend up to day 21 pi, although a sudden decrease occurred on day 28 pi (Figure 4B). However, only the Sp756.19 strain displayed similar TCID_50_ values as the reference Ss160.03 (increasing from 10^4^ TCID_50_/g on day 14 pi to 2 x 10^5^ TCID_50_/g at 28 dpi) while the titre of strain Sp939.20 was around 2 logs lower (on average 10^4^ vs 10^6^ TCID_50_/g on days 21 and 28 pi, respectively; Figure 4C). The other pilchard strain Sp997.20 showed an RNA1 copy number similar to that of Ss160.03 on days 7 and 14 pi but then displayed a significant decrease (*p* value < 0.05). The titre from day 14–28 pi was 2 logs lower than the reference strain (on average 10^4^*vs* 10^5^ – 10^6^ TCID_50_/g). Finally, as observed at 22°C, the RNA1 load of the mackerel strain Ssc1002.21 was significantly lower than that of Ss160.03 throughout the experiment, and TCID_50_ values were below the LoD. To better understand the low yield of this strain, a 1-week-analysis of the viral replication in the sole brain was conducted (Trial III). The mackerel isolate was detected in the sole brain at 2 dpi (1 day later than the sole strain) and showed an almost steady RNA1 copy number throughout the experiment, whereas the Ss160.03 load increased exponentially (1.6 logs difference between both strains at 7 dpi, Figure 4D).

**Figure 5:**
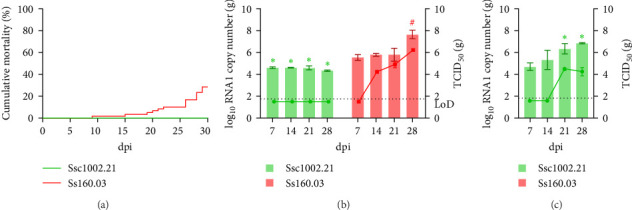
In vivo trials conducted at 15°C in Senegalese sole (Trial III) and turbot (Trial IV). Cumulative mortality (A) and viral load (B) recorded in sole and viral load in turbot (C). The viral load was expressed as log_10_ RNA1 copies: bars; and log_10_ TCID_50_/ml: line. Results are presented as the mean ± SD of the three replicates per condition. Asterisks (*⁣*^*∗*^) represent statistically significant differences regarding the reference Ss160.03 strain at each time point (*p* value < 0.05).

At 15°C (Trial IV), no mortalities were recorded in sole infected with the mackerel strain, whereas the reference Ss160.03 caused death in 28% of infected individuals (Figure 5A). According to these results, the RNA1 load of the mackerel isolate was low and stable throughout the experiment (on average 10^4^ RNA1 copies/g; Figure 5B), and although it was recovered in E-11 cells from all sampling points, TCID_50_ values were below the LoD (Figure 5B). The RNA1 load in sole infected with the homologous strain was 1 log higher than the mackerel strain until day 21 (on average 10^5^ RNA1 copies; *p* value < 0.05) and then showed a significant increase (10^8^ RNA1 copies; *p* value < 0.05). Similarly, titration showed an upward trend from 10^4^ on day 14 pi to 5 x 10^6^ TCID_50_/g on day 28 pi (Figure 5B).

The experimental infection in turbot at 15°C (Trial V) was performed only with the mackerel isolate due to the lack of reassortant strains isolated from the homologous species. Although no deaths were recorded, the virus was able to replicate as shown by the significant increase in the genomic load during the course of the experiment (from 5 × 10^4^ copies on day 7 pi to 7 × 10^6^ copies on day 28 pi; Figure 5C). In addition, infective virus was recovered in E-11 from all sampling points, and although at 7 and 14 dpi TCID_50_ values were below the LoD, the average titre detected on days 21 and 28 pi was 10^4^ TCID_50_/g.

VER signs were observed from day 5 pi onwards in sole infected at 22 and 18°C, including swirling and resting belly up at the bottom of the tank, although no reduction in food intake was detected. Neither turbot nor sole infected at 15°C showed VER signs. In addition, neither mortalities nor the NNV genome were detected in mock-infected fish.

### 3.3. Prediction of the Secondary Structure of 5′ and 3′ NCR of RNA1 and RNA2 of Strain Ssc1002.21

**Figure 6:**
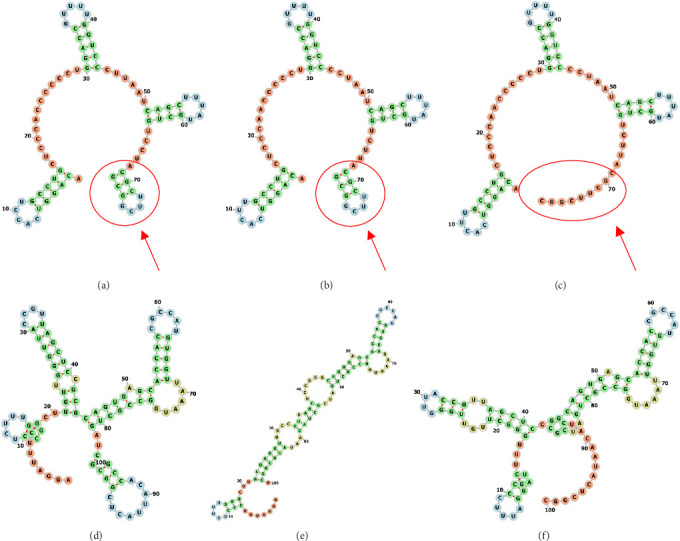
Secondary structure prediction of RNA1 and RNA2 3′-NCR. Complete 3′-NCR of RNA1: the reference strain SGWak97 (A), the sole strain Ss160.03 (B) and the mackerel isolate Ssc1002.21 (C); the last 100 nt of 3′-NCR RNA2: the reference strain SJNag93 (D), the sole strain Ss160.03 (E) and the mackerel isolate Ssc1002.21 (F). Green: stem (canonical helices); Blue: hairpin loops; Orange: unpaired region. Arrows indicate differences in the structure between strains.

As we had already sequenced the coding region of both genomic segments of the four wild strains [13], we decided to analyse the NCRs of the mackerel strain Ssc1002.21 because of its differential behaviour observed both in vitro and in vivo. Our results indicated that both genomic segments were 1 nt shorter than those of the reference strains (SGWak97 and SJNag93, for RNA1 and RNA2, respectively). In RNA1, the absence of this final nt (G3105) at the 3′ end involved a significant change in the predicted secondary structure, as Ssc1002.21 lacks the terminal loop observed in the SGWak97 and Ss160.03 strains (Figure 6A–C). In RNA2, the lack of the final nt (G1421) in the 3′ termini, together with a few substitutions, resulted in a different prediction of the secondary structure for each strain (Figure 6D–F). The mackerel strain Ssc1002.21 shares a nt change in position 58 of the NCR with the sole strain Ss160.03 (T58) with respect to the parental SJNag93 (C58) and shows an additional change with respect to the Ss160.03 strain in position 146 (Ssc1002.21: G; Ss160.03: A). However, in the 5′ NCR only one difference was observed in the RNA1 segment (position 56, C) with respect to SGWak97 and Ss160.03 (T) and does not affect the predicted secondary structure.

## 4. Discussion

NNV reassortant (RGNNV/SJNNV) strains isolated from farmed and wild fish in Southern Europe present differences in both capsid and polymerase proteins with respect to the original genotypes [6, 7, 13, 24, 25]. Some of these changes potentially affect viral fitness since they are included in the region of the RdRp responsible for temperature adaptation (positions 1–445) [8, 26, 27]. Others, located in the protrusion domain (P-domain; positions 221–338) of the Cp, have been demonstrated to be molecular virulence markers or host specificity determinants [9–11]. Therefore, the objective of the present study was to analyse the effect of temperature on the replicative fitness of 4 wild NNV reassortant isolates obtained from pilchard and mackerel [13] as well as their pathogenicity to the marine fish farmed in the same area (sole and turbot).

In vitro replication assays have shown a significantly different replication pattern of the mackerel isolate Ssc1002.21 regarding the reference sole strain Ss160.03, consisting of a delayed replication at both 25 and 20°C coupled with a higher production of viable particles at 15°C. These results contrast with previous studies that show that the optimal temperature for reassortant in vitro replication is 25°C and that replicative fitness is poorer at lower temperatures [8, 27]. The mutations found in the RdRp of the mackerel strain can account for this surprising thermolability, notably at position 237, that was not detected in any other reassortant [13]. This mutation involves the substitution of a hydrophilic amino acid (serine) by a positively charged one (arginine) in the region responsible for temperature adaptation, and specifically, in a transmembrane domain responsible for targeting the RdRp to the mitochondria so as to form the replication complex [26–28]. The temperature at which RNA viruses replicate may alter the biophysical properties of viral RNA and proteins and affect their interactions with host proteins [29, 30]. Therefore, Arg237 may favour the formation of the replication complex at mid-low temperatures. In addition, this isolate exhibits changes in the NCR sequence of both genomic segments, including mutations that have not been observed to date in any other reassortant and the lack of the last nucleotide at RNA1 3′termini, that lead to a different prediction of the secondary structure of the 3′ ends of both segments. The 3′ end of the RNA2 molecule plays a critical role in the regulation of NNV replication through interaction with RNA1 and probably with host cellular proteins [23]. We could hypothesise that the observed differences in the NCRs may also contribute to the different replication of the mackerel isolate at low temperature, as it has been reported that the 3′ termini of West Nile Virus can modulate replication in a temperature-dependent manner [30].

In vivo trials were conducted at 22°C, which is optimal for RGNNV/SJNNV in vivo replication [14], at 18°C, because this is the temperature at which sole is commonly reared in farms in our area, and at 15°C, a similar temperature to that recorded in the waters from which pilchard and mackerel isolates were obtained [13]. Cumulative mortality in sole was low–moderate and lower than that provoked by the homologous strain, regardless of temperature. Although reassortant strains are highly pathogenic for two species (sole and gilthead sea bream), differences in mortality levels caused by homologous and heterologous NNV isolates in different fish species have already been described [31–33]. The wild strains also showed a limited replication in sole that may be due to their low ability to bind sole cell receptors. Virus–receptor interaction is a critical step in viral infection and plays a major role in pathogenesis [34, 35]. Although the wild strains share substitutions at positions 247 and 270 of the CP present in the sole strain, which were considered to favour interaction with the cells of this host species [10, 36], the wild strains show 1–2 additional changes in the protruding domain. Among them, positions 237 (Tyr→Hys) and 255 (Ala→Val) of the mackerel and pilchard strain Sp939.20, respectively, are exposed on the Cp surface [37]; thus, we can hypothesise that these amino acids may be involved in the inefficient attachment to sole cell receptors favouring instead the interaction with those of the fish species they were isolated from. Additionally, a reduced interaction between the NCRs of both RNA segments of these isolates and sole cell proteins during viral replication could not be ruled out.

A remarkable finding from the in vivo experiments is the performance of the mackerel isolate at 15°C, temperature, which showed the best in vitro production of all the strains tested. In the sole, a plateau in viral replication was observed and, although viable particles were recovered in cell culture from all experimental groups, titration was not possible. Therefore, these results seem to support our hypothesis that the interaction with host proteins, not only with cell receptors but also with proteins involved in RNA translation and post-translational modifications [38, 39], could account for the scarce replication in sole. However, viral load in turbot increased exponentially and reached high values (both for RNA1 copies and TCID_50_). These results contrast with the low replication shown by the sole strain in this species at this temperature [31]. Our results indicate that adsorption to turbot cell receptors is suitably produced, suggesting that these molecules might be similar to those of mackerel cells. However, no mortalities were detected, which may be attributed to other factors, probably related to the fish immune response. In fact, an exacerbated immune response has been associated with NNV pathogenesis in sole [40, 41].

In conclusion, this study indicates that the mackerel strain has adapted to replicate at mid-low temperatures. This acclimatisation raises concern about the capacity of this strain or similar strains occurring in the wild to compete with or even replace BFNNV strains commonly detected in cold water species in the North Atlantic Ocean [1, 42]. Therefore, it is necessary to stress the importance of identifying and characterising the NNV strains naturally occurring in the wild and their virulence to farmed fish to correctly assess the potential risks associated with their introduction into fish farms or their spread to new areas.

## Figures and Tables

**Table 1:** GenBank accession number (Acc. No) for the wild strains, the reference sole strain Ss160.03, and the reference genotypes.

Strain	Acc. no for RNA1	Acc. no for RNA2
Sp756.19	ON745747	ON745751
Sp939.20	ON745748	ON745752
Sp997.21	ON745749	ON745753
Ssc1002.21	ON745750	ON745754
Ss160.03	NC_024492.1	NC_024493.1
SGWak97	NC_008040	—
SJNag93	—	AB056572

## Data Availability

The data that support the findings of this study are available from the corresponding author upon reasonable request.
